# Supplementary material for: Modeling glioblastoma heterogeneity as a dynamic network of cell states
Source: Mol Syst Biol. 2021 Sep 16;17(9):e10105. doi: 10.15252/msb.202010105 (PMC8444284; doi:10.15252/msb.202010105)
Supplement: Supplementary file 6 — Source Data for Figure 5 [file MSB-17-e10105-s004.zip › Figure5A_sourcedata/GSEA_3071/hallmarks_stateII.GseaPreranked.1623742536692/HALLMARK_HYPOXIA.html]

Details for gene set HALLMARK\_HYPOXIA[GSEA]

|  || Dataset | state23071 |
| Phenotype | NoPhenotypeAvailable |
| Upregulated in class | na\_neg |
| GeneSet | HALLMARK\_HYPOXIA |
| Enrichment Score (ES) | -0.24298234 |
| Normalized Enrichment Score (NES) | -1.2900715 |
| Nominal p-value | 0.13368055 |
| FDR q-value | 0.45078072 |
| FWER p-Value | 0.977 |
Table: GSEA Results Summary

  

Fig 1: Enrichment plot: HALLMARK\_HYPOXIA      
 Profile of the Running ES Score & Positions of GeneSet Members on the Rank Ordered List

  

| PROBE | GENE SYMBOL | GENE\_TITLE | RANK IN GENE LIST | RANK METRIC SCORE | RUNNING ES | CORE ENRICHMENT || 1 | F3 |  |  | 13 | 1.555 | 0.0288 | No |
| 2 | MT1E |  |  | 106 | 1.033 | 0.0272 | No |
| 3 | CAV1 |  |  | 134 | 0.988 | 0.0408 | No |
| 4 | PFKP |  |  | 136 | 0.988 | 0.0609 | No |
| 5 | PIM1 |  |  | 184 | 0.896 | 0.0676 | No |
| 6 | PLAUR |  |  | 185 | 0.893 | 0.0860 | No |
| 7 | EGFR |  |  | 198 | 0.874 | 0.1010 | No |
| 8 | SERPINE1 |  |  | 299 | 0.756 | 0.0917 | No |
| 9 | MYH9 |  |  | 323 | 0.735 | 0.1011 | No |
| 10 | CAVIN1 |  |  | 339 | 0.719 | 0.1122 | No |
| 11 | AKAP12 |  |  | 349 | 0.711 | 0.1245 | No |
| 12 | KLF6 |  |  | 357 | 0.708 | 0.1374 | No |
| 13 | MT2A |  |  | 393 | 0.682 | 0.1427 | No |
| 14 | ENO1 |  |  | 501 | 0.629 | 0.1290 | No |
| 15 | COL5A1 |  |  | 556 | 0.599 | 0.1279 | No |
| 16 | SDC4 |  |  | 583 | 0.585 | 0.1335 | No |
| 17 | DUSP1 |  |  | 599 | 0.578 | 0.1417 | No |
| 18 | ERO1A |  |  | 767 | 0.510 | 0.1106 | No |
| 19 | SLC2A1 |  |  | 787 | 0.503 | 0.1162 | No |
| 20 | BCL2 |  |  | 813 | 0.494 | 0.1202 | No |
| 21 | ANXA2 |  |  | 839 | 0.488 | 0.1240 | No |
| 22 | ADORA2B |  |  | 841 | 0.486 | 0.1338 | No |
| 23 | LOX |  |  | 843 | 0.486 | 0.1435 | No |
| 24 | ACKR3 |  |  | 962 | 0.454 | 0.1235 | No |
| 25 | MAFF |  |  | 986 | 0.447 | 0.1270 | No |
| 26 | IER3 |  |  | 1010 | 0.442 | 0.1304 | No |
| 27 | TNFAIP3 |  |  | 1070 | 0.424 | 0.1244 | No |
| 28 | CDKN1A |  |  | 1088 | 0.419 | 0.1288 | No |
| 29 | BGN |  |  | 1105 | 0.416 | 0.1334 | No |
| 30 | TPI1 |  |  | 1111 | 0.415 | 0.1407 | No |
| 31 | PPP1R15A |  |  | 1284 | 0.381 | 0.1057 | No |
| 32 | LDHA |  |  | 1374 | 0.361 | 0.0910 | No |
| 33 | P4HA2 |  |  | 1398 | 0.358 | 0.0927 | No |
| 34 | TMEM45A |  |  | 1401 | 0.357 | 0.0995 | No |
| 35 | NR3C1 |  |  | 1510 | 0.339 | 0.0796 | No |
| 36 | EDN2 |  |  | 1588 | 0.326 | 0.0672 | No |
| 37 | ETS1 |  |  | 1721 | 0.308 | 0.0407 | No |
| 38 | JUN |  |  | 1725 | 0.307 | 0.0462 | No |
| 39 | PGM2 |  |  | 1783 | 0.301 | 0.0382 | No |
| 40 | SDC3 |  |  | 1908 | 0.284 | 0.0132 | No |
| 41 | TPBG |  |  | 1914 | 0.284 | 0.0178 | No |
| 42 | NFIL3 |  |  | 2056 | 0.267 | -0.0118 | No |
| 43 | JMJD6 |  |  | 2062 | 0.266 | -0.0075 | No |
| 44 | ERRFI1 |  |  | 2086 | 0.264 | -0.0078 | No |
| 45 | PGK1 |  |  | 2126 | 0.260 | -0.0122 | No |
| 46 | UGP2 |  |  | 2141 | 0.258 | -0.0104 | No |
| 47 | FOSL2 |  |  | 2216 | 0.250 | -0.0236 | No |
| 48 | PRDX5 |  |  | 2373 | -0.267 | -0.0569 | No |
| 49 | SRPX |  |  | 2393 | -0.270 | -0.0561 | No |
| 50 | DTNA |  |  | 2414 | -0.272 | -0.0555 | No |
| 51 | RORA |  |  | 2513 | -0.287 | -0.0740 | No |
| 52 | PHKG1 |  |  | 2519 | -0.288 | -0.0693 | No |
| 53 | SDC2 |  |  | 2574 | -0.297 | -0.0766 | No |
| 54 | GPC4 |  |  | 2577 | -0.298 | -0.0710 | No |
| 55 | PRKCA |  |  | 2757 | -0.327 | -0.1088 | No |
| 56 | GLRX |  |  | 2834 | -0.342 | -0.1207 | No |
| 57 | ZNF292 |  |  | 2882 | -0.352 | -0.1251 | No |
| 58 | HK1 |  |  | 2997 | -0.371 | -0.1458 | No |
| 59 | MXI1 |  |  | 3000 | -0.372 | -0.1387 | No |
| 60 | HSPA5 |  |  | 3335 | -0.468 | -0.2122 | No |
| 61 | VHL |  |  | 3364 | -0.484 | -0.2092 | No |
| 62 | WSB1 |  |  | 3499 | -0.541 | -0.2314 | Yes |
| 63 | VEGFA |  |  | 3532 | -0.560 | -0.2278 | Yes |
| 64 | FOXO3 |  |  | 3594 | -0.601 | -0.2306 | Yes |
| 65 | KIF5A |  |  | 3631 | -0.627 | -0.2267 | Yes |
| 66 | CCNG2 |  |  | 3650 | -0.648 | -0.2178 | Yes |
| 67 | PAM |  |  | 3685 | -0.671 | -0.2124 | Yes |
| 68 | CXCR4 |  |  | 3733 | -0.710 | -0.2095 | Yes |
| 69 | KLHL24 |  |  | 3743 | -0.723 | -0.1969 | Yes |
| 70 | NCAN |  |  | 3763 | -0.748 | -0.1862 | Yes |
| 71 | CDKN1B |  |  | 3812 | -0.800 | -0.1817 | Yes |
| 72 | CDKN1C |  |  | 3821 | -0.809 | -0.1670 | Yes |
| 73 | HEXA |  |  | 3902 | -0.947 | -0.1674 | Yes |
| 74 | FOS |  |  | 3911 | -0.966 | -0.1495 | Yes |
| 75 | PDGFB |  |  | 3959 | -1.084 | -0.1389 | Yes |
| 76 | NEDD4L |  |  | 3960 | -1.090 | -0.1164 | Yes |
| 77 | CSRP2 |  |  | 4000 | -1.238 | -0.1007 | Yes |
| 78 | PNRC1 |  |  | 4022 | -1.397 | -0.0771 | Yes |
| 79 | STC1 |  |  | 4026 | -1.424 | -0.0485 | Yes |
| 80 | MAP3K1 |  |  | 4032 | -1.477 | -0.0194 | Yes |
| 81 | IGFBP3 |  |  | 4058 | -1.726 | 0.0100 | Yes |
Table: GSEA details [plain text format]

  

Fig 2: HALLMARK\_HYPOXIA: Random ES distribution      
 Gene set null distribution of ES for **HALLMARK\_HYPOXIA**
